# The use of simulation to prepare and improve responses to infectious disease outbreaks like COVID-19: practical tips and resources from Norway, Denmark, and the UK

**DOI:** 10.1186/s41077-020-00121-5

**Published:** 2020-04-16

**Authors:** Peter Dieckmann, Kjetil Torgeirsen, Sigrun Anna Qvindesland, Libby Thomas, Verity Bushell, Hege Langli Ersdal

**Affiliations:** 1 Department of Quality and Health Technology, Faculty of Health Sciences, University of Stavanger, Stavanger, Norway; 2 Copenhagen Academy for Medical Education and Simulation (CAMES), Center for Human Resources and Education, Herlev and Gentofte Hospital, Borgmester Ib Juuls Vej 1, Opg. 1 - 25th floor, DK-2730 Herlev, Capital Region of Denmark Denmark; 3 Department of Clinical Medicine, Copenhagen University, Copenhagen, Denmark; 4 Stavanger Acute Medicine Foundation for Education and Research (SAFER), Stavanger, Norway; 5 Department of Research, Stavanger University Hospital, Stavanger, Norway; 6 Emergency Department, Kings College Hospital NHS Foundation Trust, London, UK; 7 The Blizard Institute, Queen Mary University, London, UK; 8 Postgraduate Medical and Dental Education Department, Kings College London, Denmark Hill, London, UK; 9 Department of Anaesthesiology and Intensive Care, Stavanger University Hospital, Stavanger, Norway

## Abstract

In this paper, we describe the potential of simulation to improve hospital responses to the COVID-19 crisis. We provide tools which can be used to analyse the current needs of the situation, explain how simulation can help to improve responses to the crisis, what the key issues are with integrating simulation into organisations, and what to focus on when conducting simulations. We provide an overview of helpful resources and a collection of scenarios and support for centre-based and in situ simulations.

## Background

Simulation has a huge potential to help managing the global COVID-19 crisis in 2020 and in potentially similar future pandemics. Simulation can rapidly facilitate hospital preparation and education of large numbers of healthcare professionals and students of various backgrounds and has proven its value in many settings [1–10]. It can be utilised to scale-up workforce capacity through experiential learning. Simulation and simulation facilitators can also contribute to the optimisation of work structures and processes. In this article, we describe this potential and share ways in which it could be utilised in healthcare organisations under pressure.

## The role of simulation in the COVID-19 pandemic

The COVID-19 outbreak is a textbook case for the use of simulation and an opportunity for simulation to play to its strengths. This was seen in previous crisis events inside and outside of healthcare [11–14]. The demands of clinical care are sensitive to errors, and the stakes are high if errors occur. The pandemic places a high personal risk for healthcare professionals themselves, potentially triggering fear of getting infected or spreading the infection to their own family members. Training in the clinical environment is risky because of the danger of contamination. Practice through simulation can reduce the cognitive load [15] of the staff involved in patient care, thereby helping to mitigate error in times of pressure and exhaustion.

The rapid onset of COVID-19 and its huge burden on resources requires coordinated action across many areas of the healthcare system including staffing, equipment supply chains, bed management, diagnostic capabilities, nursing and medical treatment, infection control, and hygiene skills compliance [6]. In terms of equipment and human resources, the demand exceeds what is available in most current healthcare systems [16, 17]. Therefore, smart and novel ways of increasing and upskilling a workforce, locating and supplying equipment, and optimising work systems are needed. Simulation can play a vital role in solving these problems, and simulation educators often possess valuable capabilities to facilitate the necessary analytical work required to match (learning) needs, content, and methods to implement effective interventions. Given the urgency of the situation, careful analysis of learning needs and simulation focus points are critical, so that procedures are followed correctly and that there is appropriate use of resources to enable effective patient care.

## Aim of this paper

The aim of the current paper is to present tips and resources for the use of simulation to make a difference in the response to COVID-19. Collectively, the authors have significant experience in analysing work systems, in interacting with leaders and clinicians at various levels, and in creating simulation activities for basic education, advanced training, and research. We also have been intensely involved in coordinating, designing, and running COVID-19 simulations and other activities for our organisations in various roles. We will draw on this expertise and our knowledge of resources to support other simulation initiatives to respond to COVID-19. We will provide examples in different areas, in order to stimulate the systematic analysis in different contexts. We do not aim for a complete overview. The current situation has accelerated many simulation-related developments, but many of the underlying principles discussed in this paper will still be relevant once the crisis is over.

## Putting plans into practice

Simulation can play a role in the response to COVID-19 on several layers: firstly, the (re-)qualification of personnel to function quickly in a variety of positions (educational focus). Secondly, simulation can also play a role to understand and optimise workflows, bottlenecks, dependencies, etc. (system focus). Finally, simulation and the related abilities of simulation facilitators can help in supporting healthcare professionals in dealing with the emotional strain of the situation (personal focus). We will unfold all of these aspects in more detail.

A beneficial first step is to consider what resources are available in terms of people, equipment, and locations. Often, simulation facilitators are trained in a systems-approach to safety, understand the importance of feedback, and are able to guide people through goal-oriented reflections. They frequently have an in-depth knowledge of the hospital structure, processes, and people. Facilitators can use their skills to help healthcare units identify key problems that need to be dealt with. They can help find solutions and connect different people and departments that would benefit from collaboration.

It is important to listen carefully and identify the key clinical issues and to design simulations for the exact problems at hand, rather than “resell” existing courses from the shelves. Even in time-pressured situations, we believe that systematic analysis of (learning) needs and careful selection of contents and methods are crucial [18–20]. Simulation should be seen as an interventive tool for training and as a diagnostic tool for the analysis of work structures and processes [21]. Findings from training sessions can help in identifying and shaping learning goals, staff preparedness, improving systems and protocols, and ultimately patient safety.

## Tips and resources

In this section, we provide reflective questions and focus points that can guide you on how to develop localised approaches to specific challenges. There are other publications providing practical guidance for simulation activities focusing on hygiene issues, putting on and removing protective gear, taking COVID-19-related history from patients and relatives, decision-making and triaging, escorting patients, and self-protection against contamination [22, 23].

Although learning needs analysis can feel like an unneeded luxury in times of crisis, it is during such time-pressured situations that the increased precision brought by a coherent, if quick, needs analysis can yield significant benefits. Table 1 provides questions and focus points that can be used when analysing the (learning) needs of departments or when observing and debriefing clinical and simulation-based work. The questions aim to facilitate thinking about connections and feedback loops between departments, people, and initiatives. For all ideas, findings, and insights, you should consider the following:

- Who in your organisation needs to know about this?
- What problems, errors, and challenges did you observe?
- What were the reasons for them?
- What can be done to avoid them or to mitigate their negative outcomes?
- What are good ideas that should be shared?
- What is the “corridor of normal performance” [26] in terms of time-on-task or variety of approach (e.g. how long does donning and doffing of personal protection equipment typically take, and how do different people go about it)?

**Table 1:** Useful questions for needs analysis and/or reflections and debriefings

**People**	• Identify all staff who have contact with patients and their relatives. Consider: o Consider knowledge, skills, and attitudes needed to act in the crisis. o All hours and days of the week. o All aspects of a hospital stay (diagnosis, treatment, administration, catering, etc.). o What should they be able to do? o Do they have the right attitude for the work they are doing? • How many people of different professions do you need for the task, and where do they need to be? o Who are your reserves (clinical staff as well as facilitators) if people get ill and how do you activate them? • Are the people involved clear about the tasks that they need to do? Do they agree with these expectations? o Where can they get task-oriented help? o Where can they get help on a personal level? o What do they do if the situation gets out of control (getting help, escaping, etc.)? o What can you do to support them with the emotional strain?
**Tasks to be done**	• What tasks in relation to diagnosis, interventions, and care need to be prioritised? o In preparation of receiving patients o During treatment o Follow-up • What are the important tasks beyond the interaction with the patient? o Administration o Infection control o Co-ordination with other people and departments • How does personal protective equipment (PPE) affect the task? o Time for donning and doffing o Are there limitations to psychomotor activities? o Is sensory input impaired (e.g. do people need to speak louder)? o Is there need for additional storage and waste space? • How do you implement the individual tasks? These aspects are inspired by the Functional Resonance Analysis Method (FRAM) [24] o What triggers an action? o What is the expected outcome of the action (e.g. more information, a treatment step implemented)? o Will the outcome of the task meet its requirements? Is the outcome of too high or low quality compared to the resources available and is it on time? o What needs to be done before you can even start the task? o What resources are needed whilst the task is running? o What is the timeframe (e.g. duration, sequence)? o What guidance is there for the task (both official and unofficial practice)? Are different sources of guidance aligned (e.g. do the guidelines reflect clinical practice)?
**Context**	• Where would patients, relatives, and healthcare professionals meet? • Will the environment support patients and healthcare professionals psychologically? • Will the environment support the task? [25] • Is all equipment available and in the right place? • How can missing equipment be found? • What is the backup plan? • Is it easy for patients, relatives, staff, and potential volunteers to find where they need to go?
**General points**	Remain vigilant for any issues that come about in relation to managing COVID-19, and consider who you should inform about any possible insights • Surprises • Misunderstandings • Different priorities and wishes between people • Agreements made and ways to find the agreements • Challenges of implementing the procedures into practice • Adaptations and refinements of procedures on the fly • Equipment needed • Be resourceful with equipment and material • Concerns of the healthcare professionals

When conducting the needs analysis, you might assume that you know the organisation quite well already, if you have already run simulation projects in your organisation. However, a rapidly developing crisis situation can change things drastically—consider, for example, the shortage of very basic equipment. Take into account that an infectious disease crisis has the potential to change some very fundamental framework conditions that you may have previously taken for granted.

Not only does simulation training provide learning at the individual level, it has an integral part to play in systems testing. Every scenario holds the potential to learn and improve on the systems level [24, 27], and simulation can be a useful tool in the development of new standard operating procedures and policies needed to respond to the COVID-19 crisis.

Table 2 provides an overview of possible focus points, learning goals, target groups, methods, and practical considerations to improve the response to COVID-19. The immediate focus point for simulation typically is seen as educational. Whilst this focus is important, it does not make use of all the potential that simulation offers. Therefore, Table 2 also provides system and personal focus [29–31]. Besides the points mentioned in the tables, it is important that participants are familiar with the general procedures and actual practices within their organisation or department and that they keep updated with any rapid developments. Simulation and debriefs also have great potential to help healthcare workers deal with the emotional stress that they might be currently experiencing [32, 33].

**Table 2:** Focus areas and ways to address them

Focus area	(Learning) goal	Target group	Simulation/education modality	Practical considerations	Implications beyond training
Educational focus					
Infection prevention for healthcare professionals	Use appropriate personal protective equipment (PPE) and clinical equipment as per most recent guidelines. Increase awareness of infection risk and highlight potential weak points in the protection. Limit use of PPE to a minimum. Optimise procedures to minimise infection risk.	All staff who may have patient or family contact	• Teaching videos • Demonstrations • E-learning • Skills training • In situ training • Peer-to-peer feedback during training and clinical work • Checklists	For PPE, consider: • Location of equipment • Opening • Donning/doffing • Controlling correct use • Hand hygiene • Disposing • Decontamination Consider how to limit PPE use due to its limited availability (e.g. re-using equipment where infection risk allows, using mock-up equipment, combining several goals in one session). Collaborate with infectious control to ensure up-to-date information is being taught.	Be aware of the time it takes to don PPE (6–7 min) in different settings (e.g. in an ambulance). Inform other departments about this timing. Consider how to disseminate learning points into the organisation including common errors observed in training, to avoid these occurring in clinical practise.
Dealing with an agitated COVID-19 patient	Ensuring identification and communication of infection status. Ensuring own safety including use of PPE and positioning rooms next to exits. Conflict management, de-escalation techniques, principles of self-defence. Testing alarm response chains and timings.	All staff who may have patient or relative contact	• Demonstration videos • Role play with simulated patients • Skills training • Physical training • Walkthroughs of rooms	Test resistance of PPE during physical activity before doffing. Are there weak points that can be strengthened and who should you inform of this (e.g. ambulance services)?	Collaboration with psychiatry is essential. Consider expert advisors as co-facilitators and set up a training rota.
Increasing staffing to meet needs	Ensure competency of all staff including those redeploying to clinical work. Be aware of what the organisation requires from staff and consider that this might range from “perfect” to “good-enough”. Consider additional COVID-19 related tasks, e.g. PPE use, screening, procedures such as airway related treatments.	Pre-graduate students Professionals with other qualifications e.g. dentists Retired ex-healthcare professionals Redeployed external healthcare professionals Volunteers	• E-learning group discussion • Pre-recorded remote lectures • Skills training with feedback • Role play • Simulation • Clinical supervision with feedback	Do sharp needs analysis: what tasks need to be done? What should your learners not do? Ensure there is agreement on ways to escalate and call for help. Attempt to create a cascading model through which information is disseminated. Consider the quality assurance of your teaching; supervisors to quality control training and clinical practise, content experts (especially infectious control), rapid communication pathways for updates and quality checks.	Ensure organisation has methods of support for staff and clear ways of getting help. Consider offering debriefs and other forms of support particularly if working with volunteers. Consider assigning a well-being supervisor. Show your appreciation in different ways (e.g. coffee and cake).
Train for making decisions in the face of uncertainty and without regular support structures	Be aware of the new nature of decision making and that support structures may have changed. Establish a way to make decisions that are satisfactory in terms of processes and outcomes. Be aware of the emotional impact on decision makers, who may do so at a level they are not used to.	All staff who will be required to make decisions that would previously have been done by more senior or experienced staff.	• Case discussions • Full scale simulations • Lectures • E-learning • Cognitive skills training, for example using the shadow box method [10]	Identify situations for specific target groups in which difficult decisions may be required. Consider the different types of difficult decisions (e.g. distributing limited resources, selecting between courses of action, when to stop a specific course of action, refusing or withdrawing life supporting treatment, dealing with continuous reprioritising, and tackling conflict).	Clarify with organisational leaders what the legal and organisational frameworks are for the decisions taken. Ask them to state their view on situations (verbal and non-verbal channels are important). Prepare a clear and approved overview: who decides what?
Ensure clear leadership and followership roles where needed	Understand the pressures they will experience, including those arising from limited resources. Prepare for their emotional stress and legitimise expression of concerns and acknowledge challenges. Develop strategies to find leaders and followers and align their roles to organisational procedures.	All potential leaders and followers	• Group discussions • Case examples • Full-scale simulation • Presentations • Sharing of previous experiences • Cognitive skills training, for example using the shadow box method [10]	Consider an approach that will cater to individual difference, including the changing preferences of leaders and followers over time. Consider how you will prepare learners for the unknown; they may not know how they will react in a crisis, and reactions between different crisis differ.	Consult with crisis intervention teams if possible. Consider local customs, policies, and procedures.
System focus					
Optimise the layout and equipment available in COVID-19 rooms/wards Use simulation to test systems and improve processes which use these locations	Define equipment and space requirements. Optimise room layout and equipment. Optimise processes within the rooms and that use the equipment.	All staff who may have contact with patient on a ward: • Health care professionals on the ward • Housekeeping • Porters • Maintenance • Volunteers	• Table top simulation • Mockups • Talk-through procedures • Ceiling cameras to record and optimise walkways that are then replayed	During feedback and facilitation, consider that the needs, values, and attitudes of different professions may differ. Ensure safety and infection control precautions are taken when borrowing and returning equipment from clinical areas.	Discuss findings with relevant departments and inform them of what does and does not work.
Optimise flow of patients through the hospital	Design procedures to minimise the risk of spreading infection by patients and healthcare professionals.	All staff who may have patient contact, as well as: • Site management • Departmental leads • Infection control	• Table top simulations • Walk-throughs	Consider walking through each element so that no parts are overlooked and potential weak areas are highlighted. Consider creating one way “streets” in patient flow to reduce infection risk.	Involve infectious disease experts. Disseminate rapidly evolving changes; remember ambulance staff, clinics referring patients to hospital.
Dealing with the lack of equipment	Identify potential sources of equipment. Determine how long and in what circumstances different equipment can safely be used.	Logistical staff e.g. • Porters • Receptionists • Staff who know supply chains and processes	• Computer simulations with spreadsheets (consider COVID-19 patients/day, rate of spread, staffing/ equipment need etc. )[28] • Physical Mock ups • Table-top simulations	Support innovative thinking. Ensure that borrowed equipment is marked so it can be easily returned, and if borrowed from a simulation centre, it is cleared for clinical use.	Consider making emergency level agreements with companies for the delivery of equipment and devices. Social media describe impressive examples.
Personal focus					
Taking care of the well-being of healthcare professionals	Consider ways to keep healthy (beyond avoiding infection). Consider ways of dealing with fear and stress. Consider situations in which PPE might lose protective properties.	All staff	• Informal sharing of experiences • Informative material • Debriefing after certain incidents • End-of-shift discussion to pass on information	This topic may generate resistance as it might be seen as less relevant. However, where a crisis may be prolonged, it can make a massive difference. Ensure dedicated well-being staff are available.	Collaborate with staff usually responsible for psychological well-being and work conditions (psychologists, chaplains, occupational health).

Be sure to teach in accordance with the organisation’s philosophy, policies, and procedures, especially as the latter two are changing rapidly in the dynamic environment of a crisis. It might be that different stakeholders assign different priorities to different areas, and facilitation skills can help to broker compromises. Whenever possible, consider whether you can capture several potential learning goals simultaneously in each scenario or training event. For instance, because healthcare professionals might worry about getting infected themselves, it might be reassuring to incorporate a short demonstration and try-out of how resilient personal protection devices can be in different movement situations at the end of a training session.

The level of ambition for the quality of simulation activities might generate discussion among stakeholders. In predicaments such as the COVID-19 outbreak, simulation activities may have to aim for being “good enough”, rather than “perfect” [34]. Many of the issues addressed in Table 2 might not be the direct target of your simulation activities, but you still might be able to trigger and support changes in the organisation.

## Collaboration between stakeholders

The unfolding crisis poses huge challenges but also opens many windows of opportunity. It is impressive to see how quickly resources are provided, borrowed, and shared when they are being used to prepare for the challenges to come. Simulation centres have a key role through the skills of their facilitators, with their connection to clinical practice and with providing educational equipment to be used in various settings. Table 3 provides some tips on how to interact with different stakeholders in and beyond your organisation [27, 39, 40]. This is also to make sure that the use of simulation across all three focus areas is aligned with the organisational philosophy, policies, and procedures and that work as imagined corresponds as much as possible to work as done [41–43]. The table emphasises how an effective communication flow can help in running relevant scenarios, by carefully collecting information from the clinic. It also shows how communication from the simulation team into the organisation can help in discovering and managing problematic system elements, or great ideas that should be shared.

**Table 3:** General tips for the interaction with your organisation

Offer your simulation services to workplace leadership teams to help them meet the (impending) crisis.	• If your team is not recruited or charged to assist the organisation, offer the organisation your services to help prepare staff and the organisation for the crisis. • Keep in mind the urgency of the situation and how this affects staff, processes, decision-making, and priorities. • Understand the value your service can provide, the limits, and how to proceed. • If accepted, establish a clear mandate to operate including scope of activities, possibilities, limits, degree of self-determination, leadership, and reporting lines (two-way). Establish mutual agreement on how to proceed. o Once active, your team will be approached for help: anticipate which requests you can handle yourself and where you draw on the help of others. o Ensure other support functions (such as infectious disease, health and safety, quality, and others listed below) receive information on the simulation/education team’s function with intent to collaborate. • Establish an over-arching coordination lead; find mutually agreeable communication between teams to avoid straining personnel/equipment; determine time-slots for training and how to share those (i.e. intranet calendar, internal social media groups, whatever works best). • Synchronous communication via telephone and face-to-face meetings (respecting necessary pandemic transmission guidelines and keeping social distance) [35] can increase the speed in which agreements are made and misunderstandings are clarified. • Keep registers of participants, ideally in appropriate existing hospital databases. • Help individual departments to build their own competence and take over responsibility for their training. • Find existing teaching material within and outside your organisation. Your organisation or other organisations have probably created material that can be useful for you. Coordinate the search to avoid wasting colleagues’ time looking for the same material. Scale other peoples’ resources to fit your needs rather than designing new systems. Also, the resources provided in this article might be helpful in this regard. • Adjust your approach to the concrete problems and resources at hand. Use different tools, think outside of the box, but keep the problems at hand in mind. • Use existing plans and protocols relevant for the situation (e.g. pandemic plans). Try them in simulation to help identify important aspects to consider, such as bottlenecks, inconsistencies, and dependencies. Inform relevant others in and beyond your organisation about your findings.
Perform a pragmatic (training) needs analysis	• Focus on the organisation’s current (and rapidly updating) overarching crisis plans, pathways, and protocols to find training needs and situations [36, 37]. Establish ways to be notified about these updates and feedback loops that you can use to report insights from putting updates into practice. • Differentiate situations where training is required from situations which might require other approaches (e.g. equipment or resource needs, work organisation) • The greater the clinical pressures and urgency, the more important it is to analyse situations swiftly but adequately. A lot of time can be lost when working correctly but in the wrong direction. • Follow agreed reporting lines for prioritising, reporting, and feeding back findings: o Identify stakeholders and feedback pathways to them with observations and suggestions for rapid improvement based on in situ simulation [38]. o Who needs to be involved, to approve, to be informed? o Also, consider “feedback of negative decisions”: why should an initiative not be implemented?
Maintain and rapidly build alliances across the organisation	• Clinical leads: o They are the gatekeepers and must manage the rapidly developing crisis and all it entails. Be aware of their workload. o Identify and communicate how simulation sessions can help them meet their needs with the crisis. o Be an active collaborator. Bring your system-oriented/human-factors/education perspectives, and help to constructively address the challenges. Together establish the potential effect simulation should have and the best way to get there. o Focus on needs of the unit/pathway where simulation will occur. • Other support initiatives and departments: o Infectious disease teams: They are in high demand, and everyone wants their input simultaneously. Be considerate and rapidly find a way to collaborate professionally: maintain/establish good relations, expectations, and find a way to work together to support staff, bringing together the best knowledge and education methods to provide efficient education. o Other profession-based education staff: How they can help in the different approaches? Consider, for example, educators from midwifery, nursing, ambulance services, educators. o Health and safety: They are important as support and resources for staff well-being and the mental and physical health of staff. These aspects will be stressed in a crisis; it is useful to remind participants of this in debriefs. o Quality improvement: Collaborate with quality improvement experts for their methods that complement repeated in situ team training for continuous improvement, for example, protocol adjustments (i.e. stroke team simulations become “patient with stroke and COVID-19”). Probe the system [5], and in collaboration with clinical senior decision makers, revise the protocol for infectious disease situations. o Administrative staff: get help in booking (educational) rooms; assist in maintaining log of attendance; registration of education/preparation activities in staff competency plans; coordination of multiple simulation activities to avoid over-load on certain departments and double-booking of simulation. Create accessible e-calendar for overview and procedures to keep them updated. o Logistical staff: procuring adequate storage for in situ equipment, organising the laundry of simulation clothing. o Media and communications department: help to promote or advertise simulation activity, both for staff and the public: “We train for your safety.”

## Preparing, running, and debriefing simulations to improve responses for COVID-19

Importantly, most simulation educators have significant training and experience that make them ideally suited to leading and working through this crisis. Do not forget what you already know. Most principles of simulation apply for the current situation. Table 4 provides some practical considerations to prepare, run, and debrief scenarios with a special focus on COVID-19. We assume here that you are working within an established simulation programme and therefore do not go into all methodological details [47–49]. Make sure that you do not introduce risks by simulating in situ [50, 51], and try to get all the help you can. Remember pre-graduate students can help too [46]. Use your experience from planning and organising debriefings, as debriefing is central to learning through simulation [52–61]. The time-pressured nature of the current situation might benefit from methods which are flexible in terms of timing [62, 63].

**Table 4:** Practical considerations for the conduct of simulation sessions

General aspects	• Comply with limitations of bringing people into the same physical location in the current situation. Consider e-learning and remote connection possibilities. • Strongly encourage participants to review the organisation’s up-to-date infectious control materials (e-learning, videos, etc.) before attendance. • Beware not to introduce risks or contagions into the simulation area, especially during pandemics (but also in non-pandemic situations) [12]. o Discard/remove training equipment, do not re-use clothes for training, ensure replacement of correct equipment in clinical areas, label training equipment as such clearly, etc.
Briefing	• Clarify routines to participants, e.g. personal protection equipment (PPE), staff placement, equipment to use, clean/non-clean staff/areas. Use the time in the briefing to clearly go through these aspects, so participants may practice them in simulation. • Highlight the importance of coordinating placement of personnel during high risk aerosol-generating procedures: o Team leader takes her/his place vocally and physically in briefing, thereby establishing leadership. o Team leader establishes explicit ground rules with team for communication in general with PPE and good use of time-outs prior to invasive/risky procedures. If using air-isolation room for infectious control, ensure staff knows how to use this, and coordination of communication is practiced if these are new skills.
Scenario	• Wear PPE (if you can use it) or mock-PPE for the simulation. This reinforces the need for teams to communicate very clearly as PPE disrupts concurrent lip reading and the understanding of facial expressions; additionally, speech audibility tends to be slightly reduced. (Video recordings might make this obvious.) • Most hospitals must conserve use of PPE for actual clinical patients: devise mock-equipment that can stimulate learning and try not to use precious PPE resources if possible, e.g. write “FFP3” onto surgical masks or use masks participants were fit-tested with. Use different coloured aprons to represent different types of gowns. • Be very aware of potential risks of spreading the infection in case of re-use of equipment (prevent re-use where this risk exists). • If observing infection control breaches, consider time-out procedures for immediate feedback; ask participants to observe, ascertain, and correct breach, then continue.
Debriefing	• Respect that most healthcare professionals are interested in correct PPE and infectious disease control activities during a pandemic. Do not forget other important human-factors issues, such as teamwork, situational awareness, and communication that might also be related to safe care [44, 45]. • Topics with likely high interest: o Task collaboration between “clean” and “non-clean” personnel. o Time-outs and clearing personnel before interventions. o Communication with PPE. o Infection control during handover and transport. o Self-protection and other technical questions (participants tend to have many of these and want robust answers) • Either follow up personally or brief other people in the organisation about critical un-resolved questions and establish feedback relevant loops • Be prepared to adjust/re-adjust to changing protocols. • Consider the timing of the debrief. Are participants going to stay for the whole session, or may they be called back to clinical service? If so, consider options such as stop-start scenario and running commentary to highlight positive and problematic behaviours. You can still do a post-simulation debrief if time allows.
Logistics	• Prior to in situ training, check the feasibility for simulation onsite (remember to check electricity outlets and other material). Be as independent as you can by bringing all the equipment needed, thereby minimising the strain on the departments in which you simulate. If your hospital is working under a major incident command structure (like it would be in the UK), you will have to get appropriate clearance (e.g. silver or gold command level authorization in the UK). • Have a clear checklist for the equipment and requirements that you cannot bring yourself and what you expect the department in which you simulate to provide (e.g. access codes for the Wi-Fi) • Consider involving pre-graduate students into your operations and logistics, as they are able to absorb some of the workload [46].

## Further material

COVID-19-related scenario scripts and a collection of other resources can be found at www.safer.net. A collection of scenarios from King’s College Hospital in London can be found as an online supplement. Further collections of supporting material are rapidly being circulated in online communities of practice and through social media. Use them when it is appropriate to do so, as it can save time during a crisis situation. As always, these resources will need to be adapted to local procedures.

## Long-term considerations

Remember to keep also the long-term perspective in mind: the crisis will subside and some sense of a normal clinical service will resume, potentially with a backlog of necessary activity that has been put on hold during the crisis. How will you, your colleagues, and the overall system get back to normal after this situation? What might “normal” might look like? It may very well be quite different than it has been before the crisis. Is there an opportunity to have a lasting effect on, for example, patient safety, the well-being of healthcare professionals, or work processes? Take the opportunity to nurture the connections with people in clinical departments—they can help to get the job done in clinical practice, especially when it needs to be done quickly. Do not forget to make some notes for the future: aspects of the crisis experience can help you to develop plans on how to use simulation to prepare organisations to respond to the unexpected and the extraordinary in the future.

## Summary of key points

- Ensure the needs of the organisation are well understood.
- Consider connections and establish feedback loops.
- Know what resources are available in terms of material, equipment, and people.
- Find the balance between providing learning and providing systems improvement.
- Be aware of the potential extra load put on learners, educators, departments, clinical units, and the organisation during a crisis.
- Consider the knowledge and skills your learners require, as well as their well-being. Help them to protect themselves and to save valuable resources.
- Spending adequate time on the analysis of the problem is an investment towards solutions that will make a difference. Balance being fast with being thorough.

## Conclusion

Simulation has great potential to help mitigate the negative effects of the COVID-19 crisis and potentially for future crisis situations. The examples and tips provided in this paper can help simulation to harvest this potential in the interest of patients, relatives, the public, and—last but not least—healthcare professionals.

## Supplementary information

**Additional file 1.** Collection of simulation scenario scripts.

## Data Availability

We do not report empirical findings as such. Therefore, no more data or materials are available.
